# Atypical juvenile hereditary hemochromatosis onset with positive pancreatic islet autoantibodies diabetes caused by novel mutations in *HAMP* and overall clinical management

**DOI:** 10.1002/mgg3.1522

**Published:** 2020-10-05

**Authors:** Hui‐Xuan Wu, Jun‐Ying Liu, De‐Wen Yan, Long Li, Xiang‐Hua Zhuang, Hai‐Yan Li, Zhi‐Guang Zhou, Hou‐De Zhou

**Affiliations:** ^1^ National Clinical Research Center for Metabolic Diseases Hunan Provincial Key Laboratory for Metabolic Bone Diseases Department of Metabolism and Endocrinology The Second Xiangya Hospital of Central South University Changsha Hunan China; ^2^ Department of Endocrinology Shenzhen Second People's Hospital The First Affiliated Hospital of Shenzhen University Shenzhen Guangdong China; ^3^ Department of Endocrinology The Second Hospital of Shandong University Jinan Shandong China

**Keywords:** *HAMP*, juvenile hereditary hemochromatosis, pancreatic autoimmune antibody, type 1 diabetes

## Abstract

**Background:**

Atypical clinical symptoms of juvenile hereditary hemochromatosis (JHH) often leads to misdiagnosis and underdiagnosis bringing ominous outcomes, even death.

**Methods:**

The whole exome was sequenced and interpreted. A literature review assisted to analyze and verify the phenotype–genotype relationships. We revealed the entire process of diagnosis, treatments, and outcome of two diabetic onset of JHH families to provide new insights for genotype–phenotype relation with novel compound heterozygous mutations in the hepcidin antimicrobial peptide (*HAMP*, OMIM: 606464).

**Results:**

Two probands were diagnosed and treated as type 1 diabetes initially because of specific symptoms and positive islet autoantibodies. Poor control of hyperglycemia and progressive symptoms occurred. Sequencing informed that the compound heterozygous and homozygous mutations c.166C>G and c.223C>T in *HAMP* caused type 1 diabetic‐onset JHH. The two patients accessed irregular phlebotomy treatments, and then, experienced poor prognosis. We summarized the process of overall clinical management of reported 26 cases comparing to our novel atypical diabetic onsets Juvenile Hereditary Hemochromatosis cases.

**Conclusion:**

It was first reported that positive pancreatic islet autoantibodies diabetes onset of JHH resulted from loss‐of‐function mutations of *HAMP*, of which the atypical JHH should be differentially diagnosed with type 1 diabetes at the onset. Early administration of phlebotomy and vital organs protection and surveillance might be important for the treatment of atypical JHH.

## Graphical abstract

It reported the positive pancreatic islet autoantibody diabetes onset JHH case. A novel compound heterozygosity mutation in HAMP was found. Early administration of phlebotomy and vital organs protection are important.

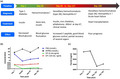

## 1 INTRODUCTION

Hereditary hemochromatosis (HH) is defined as hereditary iron overload clinical syndrome caused by a deficiency of hepcidin, including decreased production or inactivity of hepcidin–ferroportin binding (Brissot et al., 2018). According to the etiologies, inheritance and prevalence of the hemochromatosis, hemochromatosis could be divided into type 1, mutation occurs at the homeostatic iron regulator (*HFE*, OMIM: 613609) gene on chromosome 6 (Seckington & Powell, 1993), type 2A, mutation in the hemojuvelin (*HJV*,OMIM: 608374) gene on chromosome 1 (Papanikolaou et al., 2004), type 2B, mutation in the *HAMP* gene on chromosome 19 (Roetto et al., 2003), type 3, mutation of the transferrin receptor 2 gene *(TFR2*, OMIM: 604720*)* on chromosome 7 (Roetto et al., 2001), type 4, mutation in the solute carrier family 40, member 1 (*SLC40A1*, OMIM: 604653) gene on chromosome 2(Njajou et al., 2001) and type 5, mutation in the H‐ferritin (*FTH1*, OMIM: 134770) gene on chromosome 11 (Kato et al., 2001). As the most prevalent type of HH, in HFE‐associated hemochromatosis, intestinal iron uptake is modestly increased. Consequently, iron overload requires several decades to become clinically manifest (De Gobbi et al., 2002). However, in juvenile hereditary hemochromatosis (JHH), resulted from a *HJV* or *HAMP* gene homozygous mutation, iron overload is severe, and organ failure occurs before 30 years (De Gobbi et al., 2002). The loss function of *HAMP* causes inactivation of hepcidin that negatively regulates ferroportin in enterocyte and only accounts for 1/10 of JHH cases (Nemeth et al., 2004; Sandhu et al., 2018).

Individuals with JHH are rarely diagnosed at onset, especially some atypical monosymptomatic JHH cases do not have general symptoms (Goldberg, 2005 Feb 17 [Updated 2020 Jan 9]). Prominent clinical features in JHH patients are involved in multisystem and often occurred after severe iron load, including hypogonadotropic hypogonadism, cardiac arrhythmias, abdominal pain, diabetes, or glucose intolerance, and skin pigmentation (Brissot et al., 2018; Goldberg, 2005 Feb 17 [Updated 2020 Jan 9]; Roetto et al., 2003). Considering the rarity, complexity, and severity of JHH, misdiagnosis, and underdiagnosis at onset will bring ominous outcomes, even death. Therefore, clinical research on JHH caused by *HAMP* mutation, particularly atypical monosymptomatic onset manner, is vital for broadening HH spectrum and overall administrative management. Here, we summarized the phenotype, diagnosis, treatments, and prognosis of atypical monosymptomatic type 1 diabetic‐onset JHH resulting from novel compound heterozygous mutations in the *HAMP* gene cases to provide new insights and more experiences for overall clinical management.

## 2 MATERIALS AND METHODS

### 2.1 Ethics approval and consent to participate

The study was approved by the Ethics Committee of the Second Xiangya Hospital of Central South University. Informed consents were obtained from all participants for being included in the study.

### 2.2 Subjects

**FIGURE 1:**
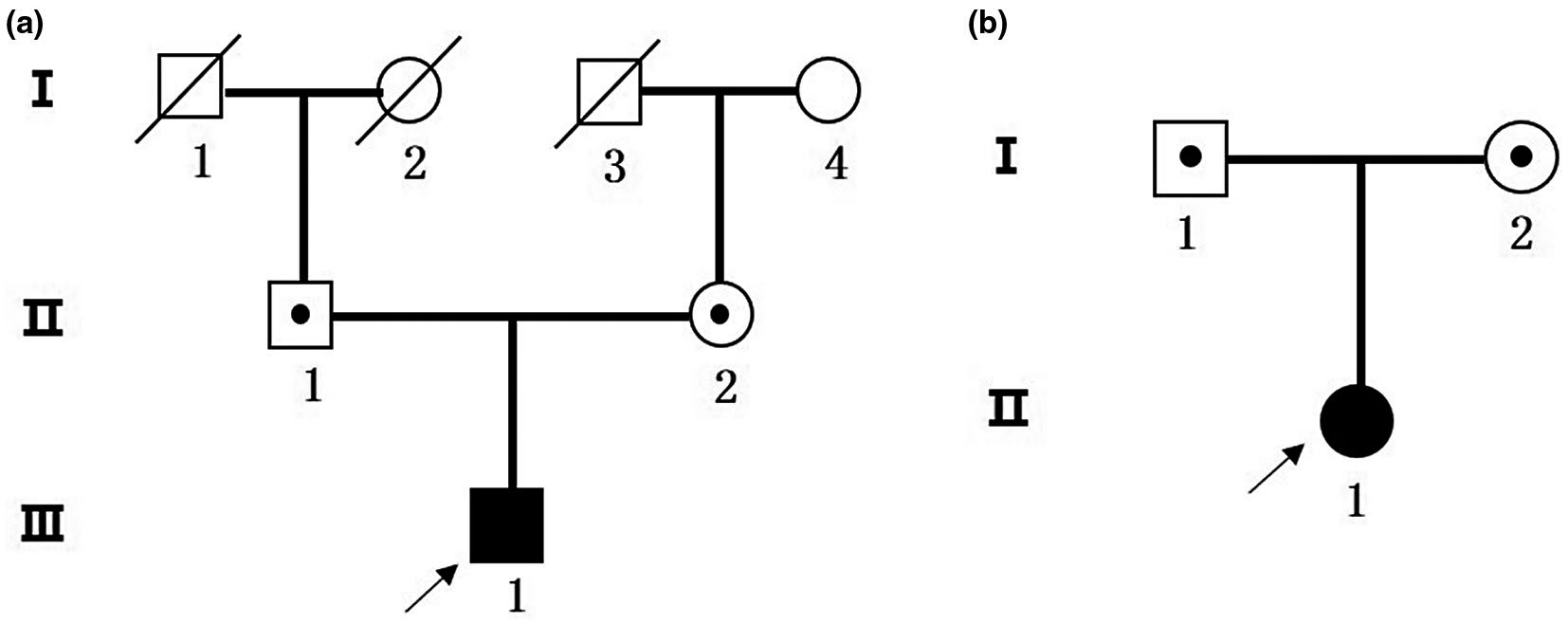
The pedigrees of two families. (a) The pedigree of family 1. (b) The pedigree of family 2. The arrow indicates the proband, squares represent male members, and circles represent female members; patients are indicated with filled symbols

The probands and their families were recruited from Department of Endocrinology, Shenzhen Second People's Hospital (Figure 1a) and the Second Hospital of Shandong University (Figure 1b). There were six subjects (probands and their parents) in all who indicated their willingness to participate. Clinical data and family history were collected by professional physicians.

### 2.3 Laboratory and imaging data collection

The blood samples were centrifuged for serum isolation, frozen, and the fasting C‐peptide, insulin and blood glucose were analyzed by the chemiluminescent method; β‐hydroxybutyric acid, tested by enzymatic colorimetric assay; HbA1c, tested by ion‐exchange HPLC with the normal range of 3.9–6.1%. Urinary ketone was tested with dry chemistry test paper. The isolated serum was sent to the Second Xiangya hospital of Central South University for further screening of zinc transporter 8 antibodies (ZnT8A), islets cells antibodies (ICA), glutamic acid decarboxylase antibodies (GAD), islet antigen type 2 (IA2), and insulin (IA) autoantibodies. The concentration of pancreatic autoimmune antibody was detected by radioligand binding assay (RLA) (Huang et al., 2012). Serum iron, unsaturated iron binding capacity were detected by colorimetry. In addition, the probands underwent electrocardiography, cranial, abdominal, and cardiac ultrasound as well as MRI, dual energy X‐ray scanning; and the following functions were tested: renal, liver, coagulation, sexual hormone, electrolytes, and lipids. Follow‐up was performed once a month for 6 months.

### 2.4 Genetic testing and analysis

Genomic DNA was extracted from peripheral blood samples taken from probands and their parents. The DNA was sent to Beijing Kangso Medical Inspection for whole‐exome sequencing. After the DNA being fragmented, the library was constructed, and then, the target fragment to be sequenced was obtained by hybridization. Next, the target fragment was enriched by PCR amplification and the products were purified and quantified. It was sequenced using Illumina's NextSeq500 sequencer to obtain the raw data. The raw data were converted into recognizable base sequences by CASAVA (1.8.2) software. Reads were mapped to the human reference genome sequence (assembly GRCh37/hg19) using the Burrows‐Wheeler Alignment Tool. Single nucleotide variants, insertion, or deletion were detected using the Genome Analysis Toolkit.

The variants sites, types, frequencies, and other information were obtained through databases and transcript annotation databases, such as 1000 Genome, Human Gene Mutation Database (HGMD), dbSNP, the Exome Aggregation Consortium (ExAC), Ensembl, UCSC, RefSeq, and Consensus CDS. The impacts on protein structure and function of variants were predicted using different mutation predictor software like Mutation Taster (www.mutationtaster.org), PolyPhen‐2 (genetics.bwh.harvard.edu/pph2), and SIFT (sift.bii.a‐star.edu.sg). The structure of protein was predicted by PSIPRED (http://bioinf.cs.ucl.ac.uk/psipred) and Phyre2 (www.sbg.bio.ic.ac.uk/phyre2). Conservation of amino acid change was analyzed by Clustal Omega sequence alignment tool and calculated by means of the WebLogo service. The *HAMP* (NM_021175) variates from probands and their relatives were amplified by PCR and sequenced by TsingKe Biological Technology (Beijing, China) with Sanger sequencing.

The candidate variants were interpreted with clinical symptoms and signs according to Standards and Guidelines for the Interpretation of Sequence Variants: A Joint Consensus Recommendation of the American College of Medical Genetics and Genomics and the Association for Molecular Pathology (Richards et al., 2015).

### 2.5 Literature review

We conducted a literature review using PubMed and Web of Science to identify all reported cases of genetically confirmed JHH caused by mutations in *HAMP* until Mar. 2020. From the available issues, data regarding the symptoms at JHH onset, phenotype, genotype, demographic, and treatments of patients were collected. The data were rechecked, and the validity of the data was verified by a second investigator. Patients were enrolled if they were homozygous, heterozygous, compound heterozygous, or complicated mutations in *HAMP*.

## 3 RESULTS

### 3.1 Clinical findings and diagnosis

The proband from family one (Figure 1a) III‐1 (1‐III‐1), a 29 years old man, of 169 cm height and 69 kg weight (BMI 24.2), was hospitalized because of fatigue, polydipsia, and polyuria for 2 weeks on 25 Aug 2017 (Day 1, Figure 3a). He had ketoacidosis owing to pH 7.26, fasting blood glucose 28.36 mmol/l, urinary ketone ++, low concentration of C‐peptide (fast C‐peptide 0.5 ng/ml, postprandial 0.56 ng/ml), HbA1c 12.3%, and positive pancreatic autoimmune antibodies, including ICA and IA–2A. The proband from family two (Figure 1b) II‐1(2‐II‐1), a 34‐year‐old woman was hospitalized because of weight loss, polydipsia, and polyuria for decades on 25 Sep 2007. Her fasting blood glucose was up to 10 mmol/l and the concentration of C‐peptide was low (fast C‐peptide 0.04 ng/ml, postprandial 0.06 ng/ml), but negative pancreatic autoimmune antibodies. We did not find significant abnormalities in liver kidney function, abdominal B‐mode ultrasonography, and electrocardiogram. According to ADA diagnostic criteria (American Diabetes, 2020), the two probands were diagnosed as type 1 diabetes. 1‐III‐1 accepted fluid infusion and insulin injection (Day 1, Figure 3a). Fasting blood glucose levels decreased but postprandial blood glucose levels were still high and C‐peptide decreased from 0.5 ng/ml to <0.05 ng/ml after 1 month of treatment with insulin (Day 36, Figure 3a). One month after insulin therapy, blood glucose levels were still poorly controlled. 2‐II‐1 also received treatment of insulin for diabetes, but she got poor blood glucose control.

**FIGURE 2:**
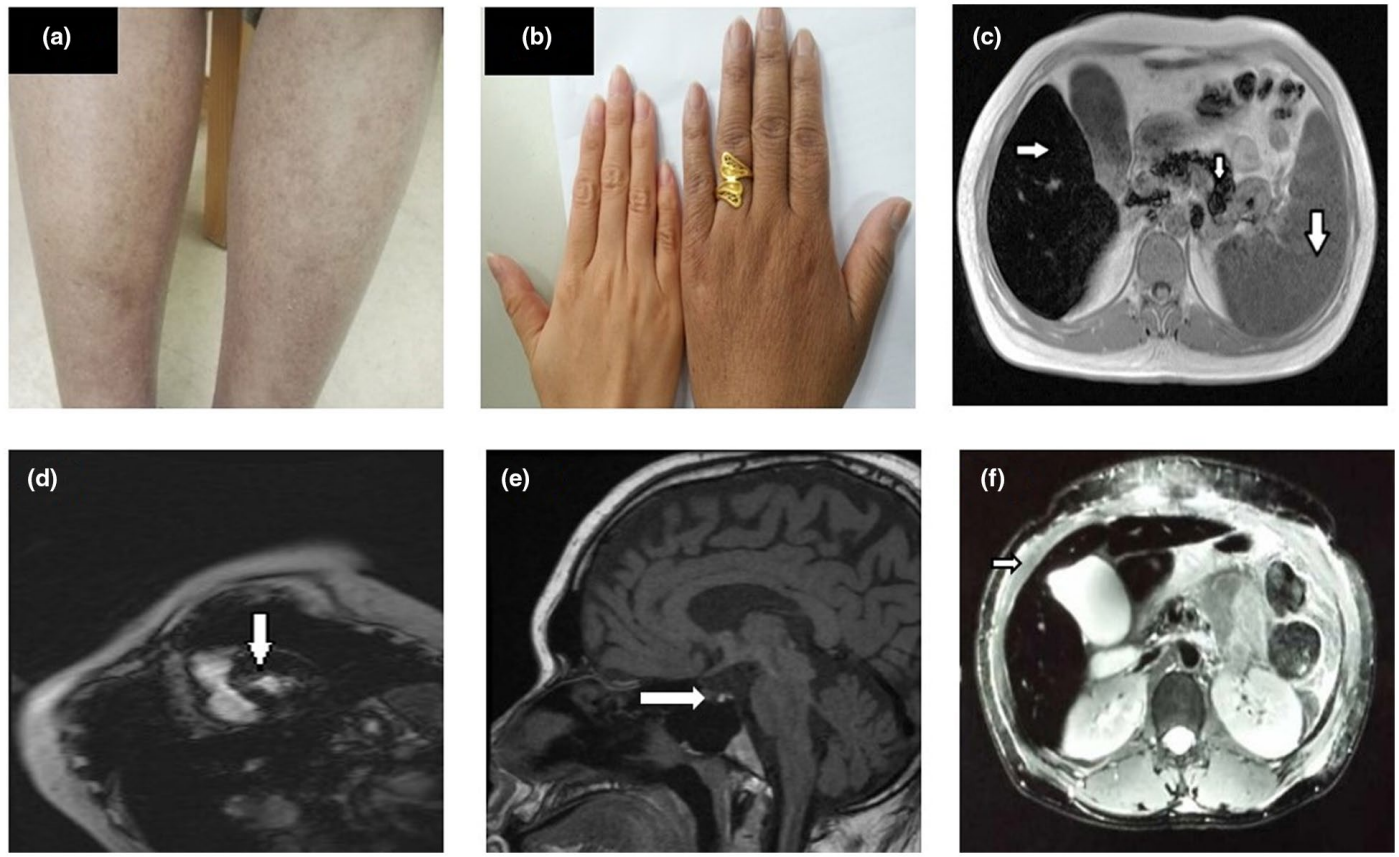
Focal skin pigmentation on limbs and MRI scan for two cases. (a) The slight pigmentation of the anterior tibia in 1‐III‐1. (b) The pigmentation of the left hand of 2‐II‐1. (c–e) The abnormal deposition of iron in the liver, myocardium, and pituitary, respectively, by MRI in 1‐III‐1. (f) The abnormal deposition of iron in the liver in 2‐II‐1

**FIGURE 3:**
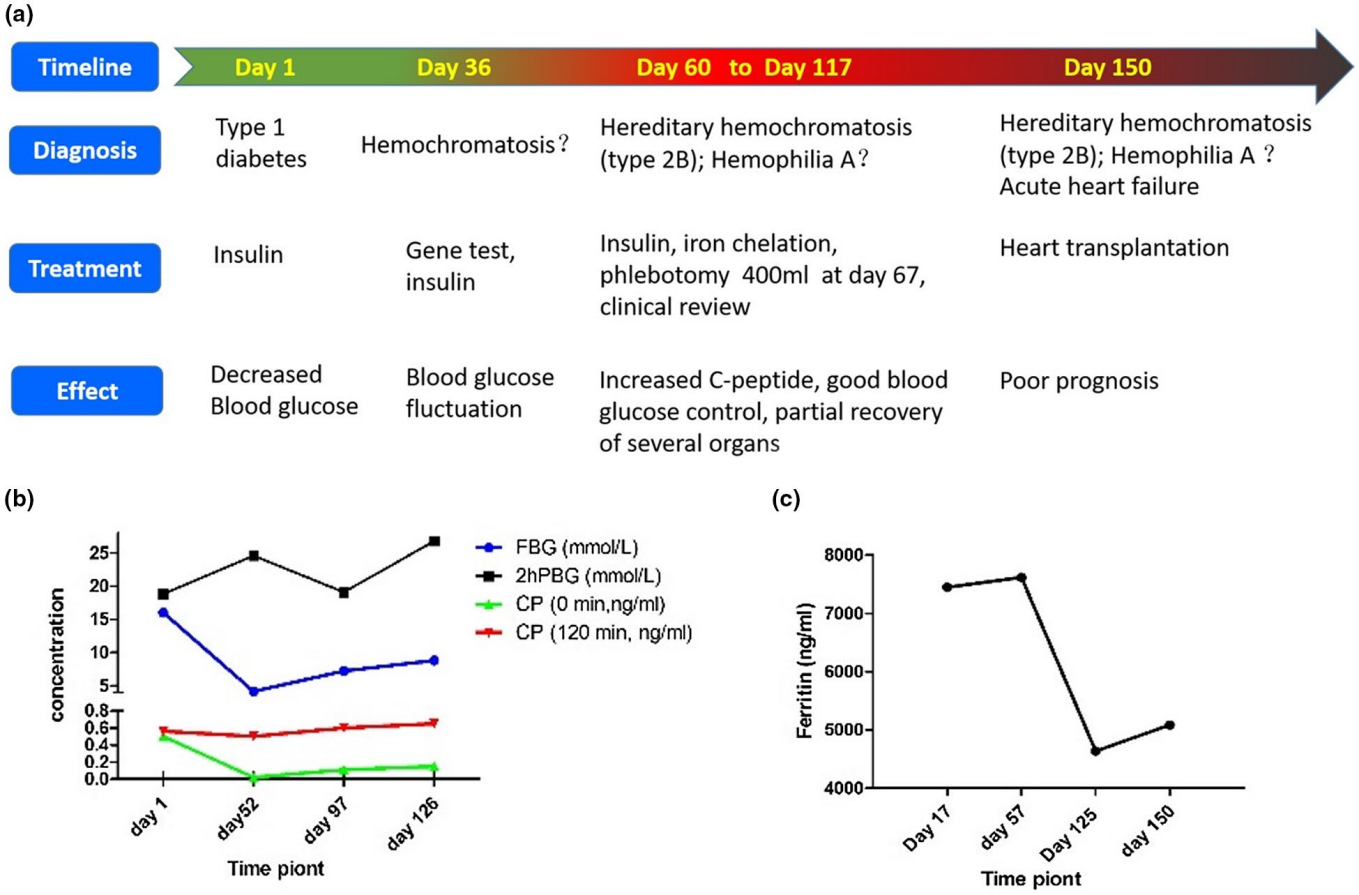
1‐III‐1’s hemochromatosis treatment in accordance with chronological order and prognosis. (a) The timeline of diagnosis and treatment of 1‐III‐1. (b) Changes in blood glucose and C‐peptides. (c) Changes in serum ferritin levels

Two cases were rehospitalized because of poor control of hyperglycemia as well as new symptoms. From Sep. to Oct 2017 (Day 35 to Day 60–117, Figure 3a), 1‐III‐1 was detected with pancytopenia several times: red blood cell 3.89 × 10^9^/L, hemoglobin 113 g/L, white blood cell 2.92 × 10^9^/L, platelet 40 × 10^9^/L. Bone marrow puncture found the number of megakaryocytes increased, accompanied by maturation disorder. In Endocrine system, 1‐III‐1 suffered from decreased ejaculatory function and erectile dysfunction, the sexual hormone and GnRH exciting test indicated hypogonadotropic hypogonadism (before GnRH irritation: FSH 0.43 IU/L, LH 0.51 IU/L; after GnRH irritation: FSH 0.57 IU/L, LH 0.76 IU/L). Duel energy X‐ray showed osteoporosis because of low Z‐score of total hips (−3.3). In May 2015, 2‐II‐1 suffered impaired liver function, including elevated alanine aminotransferase 98 U/L, aspartate aminotransferase 63 U/L, alkaline phosphatase 179 U/L, and γ‐glutamyl transpeptidase 162 U/L. She was also diagnosed as hypothyroidism because of high TSH 25.51 uIU/ml and low FT4. The ultrasound showed increased thyroid volume and uneven echogenicity. Besides, physical examination revealed bruises, slight focal pigmentation (Figure 2a) and xerosis cutis on the 1‐III‐1 limbs and interphalangeal joints, and slight pigmentation on the 2‐II‐1 hands (Figure 2b). Multiorgan involvement indicated clinical syndrome rather than type 1 diabetes. Further testing showed high ferritin level, 7 515 ng/ml for 1‐III‐1 and 26 719 ng/ml for 2‐II‐1. The transferrin saturation was 91.7% in 1‐III‐1. Their abdominal B‐mode ultrasonography showed splenomegaly and no significant change in liver. But MRI scan showed low signal (black matter lesion) in the liver, spleen, myocardium, and pituitary (Figure 2c–e) in patient 1‐III‐1, low signal (black matter lesion) in the liver and pancreas (Figure 2f) in patients 2‐II‐1, indicating abnormal deposition of iron.

### 3.2 Genetic mutations description and analysis

According to the clinical, imaging, and biological data, two patients could be clinically diagnosed as hemochromatosis (P Brissot et al., 2018). Genetic testing was explored. To 1‐III‐1, the whole‐exome sequencing results indicated a compound heterozygous mutation in *HAMP*. The c.166 C>G mutation was inherited from his father and the c.223C>T mutation from his mother. His parents did not manifest hemochromatosis clinically. Furthermore, the proband inherited c.6724G>A mutation in the *F8* gene on the maternal X chromosome, which might be pathogenic to hemophilia A (Tuddenham et al., 1994). To 2‐II‐1, the whole‐exome sequencing results indicated a homozygous mutation c.166 C>G in *HAMP* which was inherited from his parents. The pathogenic variants in *HFE*, *HJV*, *TFR2*, *SLC40A1*, *and FTH1* were not found in these two cases.

**FIGURE 4:**
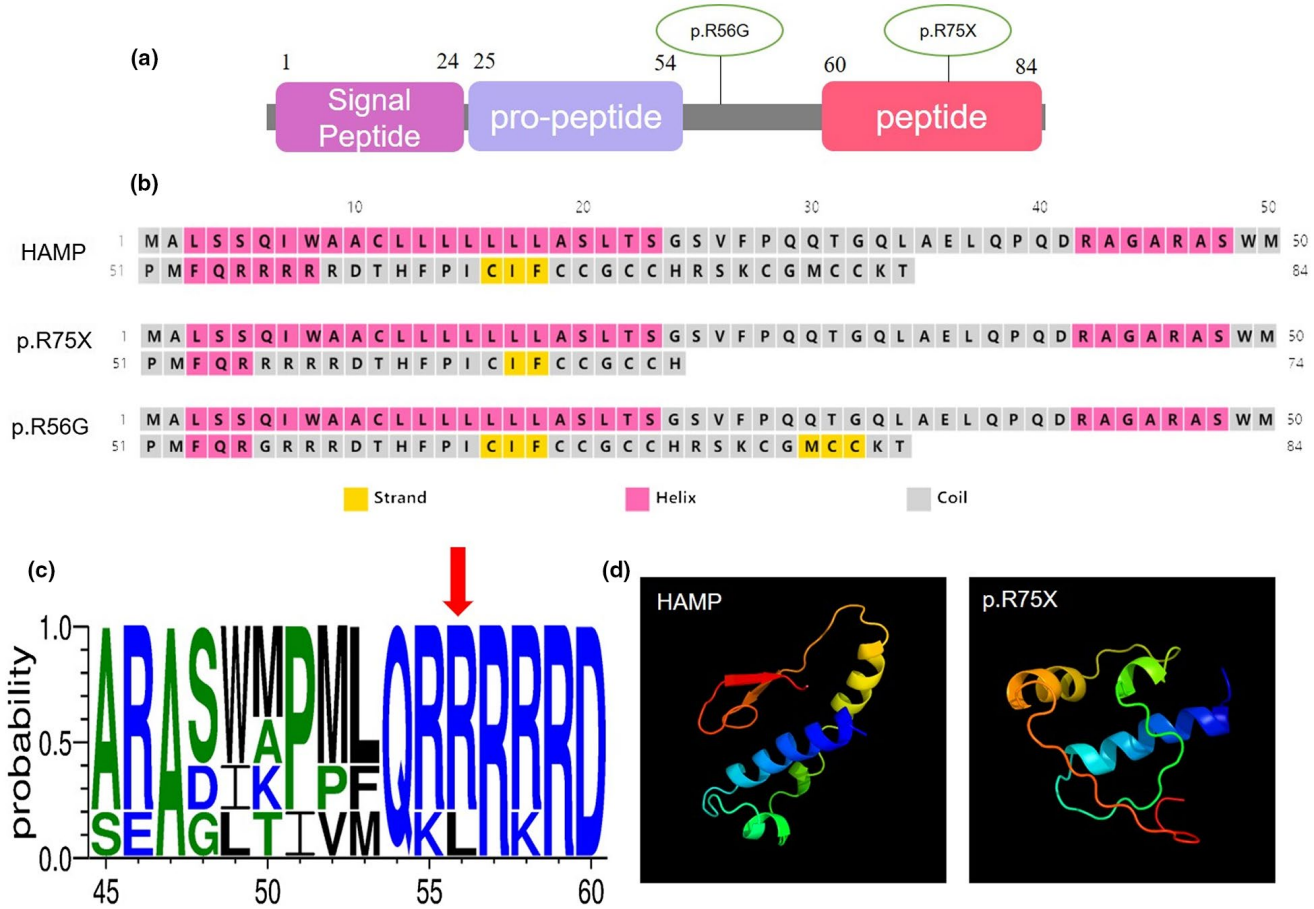
In Silico Prediction of the HAMP Mutations. (a) Schematic representation of the domains of the HAMP protein and position of the identified alteration. (b) Analysis of HAMP and variants second structure. (c) Amino acid conservation at position of HAMP Arg56. (d) The 3D structure of HAMP and p.R75X

The base change from C to G at position 166 causes the substitution of Arginine 56 with Glycine (p. Arg56Gly). The PolyPhen‐2 predicted missense mutation probably changing the protein secondary structure (Figure 4b) with a score of 0.782 and Asp56 (Figure 4c) appears to be highly conserved. The homozygous base change from C to T at position 223 causes the substitution of Arginine 75 with terminator (p.Arg75Ter) resulting in nine amino acid deletion in hepcidin (Figure 4d) which was identified no intact or partial hepcidin expression in patient's serum and urine (Hattori et al., 2012). According to classification of ACMG for assessing the pathogenicity of different variants, the c.223C>T of *HAMP* are “pathogenic” as it is the null variant in which loss‐of‐function (LOF) is a known mechanism of hemochromatosis (PVS1), absent in population data bases (PM2), recessive disorder detected in *trans* (PM3), computational evidence showed deleterious effect (PP3), and highly specific disease phenotype (JHH) with single gene (*HAMP*) (PP4). The c.166C>G of *HAMP* are “likely pathogenic” as it is absent in population data bases (PM2), recessive disorder detected in *trans* (PM3), computational evidence showed deleterious effect (PP3), and highly specific disease phenotype (JHH) with single gene (*HAMP*) (PP4).Thus, according to ACMG the two missense mutations of *HAMP* has at least two moderate and two supportive evidences of pathogenicity, fulfilled the criteria of ACMG for “pathogenic” or “likely pathogenic” variant. From the clinical, biological, genetic, and imaging data (Brissot, 2016), the two patients were diagnosed with type 2b juvenile hereditary hemochromatosis.

### 3.3 Hemochromatosis treatments and prognosis

Following the diagnosis of hemochromatosis, 1‐III‐1 was treated with deferoxamine, an iron chelation treatment, from day 60 and phlebotomy once on day 67 (Figure 3a). Consequently, there was relief from the symptoms of fatigue, polydipsia, polyuria, and anejaculation. The patient's blood cells increased and C‐peptide slightly increased (Figure 3b). The concentration of ferritin decreased from 7580 to 4062 ng/ml (Figure 3c). On day 117, he suffered abdominal distension, and then, was diagnosed of severe acute heart failure. His ejection fraction (EF) decreased to 34% compared with 58% at the beginning (Table S1). He underwent a heart transplant on day 150 (Figure 3a) but the prognosis was very poor. The hematoxylin‐eosin staining of myocardial showed iron (hemosiderin granule) deposition in cardiomyocytes. He died of severe postoperative infection.

2‐II‐1 received treatment of insulin for diabetes, levothyroxine for hypothyroidism, and polyene phosphatidyl choline for hepatic protection. But she got poor blood glucose control. After we made a clinical diagnosis of hemochromatosis, she received deferoxamine for iron chelation and refused phlebotomy. Her prognosis was also poor. Finally, she died of liver failure.

### 3.4 Literature review

There were 26 cases reported worldwide who were diagnosed of *HAMP*‐JHH with or without *HFE*, *TFR2* mutations. The main clinical manifestations of *HAMP*‐JHH are hepatic involvement, hypogonadism, skin pigmentation, hyperglycemia, cardiomyopathy, arthropathy, osteoporosis, splenomegaly, primary hematological abnormality, and hearing loss. Hepatic involvement and hypogonadism are hallmark features of *HAMP*‐JHH as the frequency of them are 75% and 61%, respectively (Table 1). There are 16 male patients and eight female patients, and most of them suffered hepatic involvement and hypogonadism initially (Table S2). From the reported cases, *HAMP*‐JHH preferentially occurred in Europe (17/26). Interestingly, all patients with mere *HAMP* mutations, whether intronic or exonic, have an onset age of less than 30 years. *HAMP* with *HFE* or *TFR2* mutations (complex mutations) patients have a later onset age. The characteristics and distribution of onset symptoms in the two groups were also inconsistent, and the onset of symptoms in the mere *HAMP* mutations group seemed to have a more tendency of severity, which needed to be supported by more data (Figure S1).

**TABLE 1:** Clinical symptoms of *HAMP*‐JHH

Clinical symptoms	Reported cases n = 26	Our study n = 2	Frequency (#/28)
Hepatic involvement	19	2	0.75
Hypogonadism	16	1	0.61
Skin pigmentation	10	2	0.43
Pathoglycemia	7	2	0.32
Cardiomyopathy	8	1	0.32
Arthropathy	7	0	0.25
Osteoporosis	2	1	0.11
Splenomegaly	1	2	0.11
Primary hematological abnormality	1	1	0.07
Hearing impairment	1	0	0.04

Note

Pathoglycemia including diabetes and impaired glucose tolerance (IGT).

## 4 DISCUSSION

The *HAMP* gene encodes hepcidin, an 84 amino acid protein, which regulates ferroprotein in enterocytes. It contains a 24‐residue N‐terminal signal peptide that is subsequently cleaved to produce pro‐peptide. Pro‐peptide is processed to produce a mature 25‐amino acid hepcidin with compact β‐sheet and hairpin loop elements from disulfide bonding (Jordan et al., 2009; Park et al., 2001) (Figure 4a). The homozygous mutation of *HAMP* can cause type 2b HH or JHH (Papanikolaou et al., 2004). Compared to *HFE* and *TFR2*‐HH, JHH carries a greater risk of heart attack, skin change, liver fibrosis, and hypogonadism (Sandhu et al., 2018), allowing easier diagnosis. However, two Chinese patients in the current report had ketone‐prone type 1 diabetes‐onset JHH. Apart from diabetes, no obvious clinical symptoms were found at the onset of JHH, and no abnormalities were found in abdominal B‐mode ultrasonography or electrocardiogram which was different from patients’ clinic spectrum reported worldwide. This led to misdiagnosis and delay of therapy. Therefore, we investigated further and discovered poor blood glucose control, decreased fasting C‐peptide, increased ferritin, liver enzyme, and pancytopenia, which allowed us to modify the diagnosis as hemochromatosis. In 1‐III‐1, considering the cytotoxicity of hyperglycemia, pancytopenia was easily ignored at first examination, which we should have paid more attention to.

Through genetic analysis, we found 1‐III‐1 owned heterozygous c.166C>G (p.R56G) mutation from his father and a heterozygous c.223C>T (p. R75X) mutation from his mother, 2‐II‐1 owned homozygous c.166 C>G (p. R56G) mutation from her parents. We made an in silico prediction of the secondary structure of p. R75X and p. R56G and the 3D structure of p. R75X *HAMP* using software PSIPRED (Jones, 1999) and Phyre2 (Kelley et al., 2015) (Figure 4b,d). The 56‐amino acid residues change from R to G occurs in a penta‐arginine (55–59) basic domain, which is highly conserved (Figure 4c) and probably the pro‐hormone convertase cleavage sites in human (Park et al., 2001). One study found that c.223C>T (p. R75X) is a nonsense mutation which can cause iron overload by impairing the hepcidin system in juvenile hemochromatosis patients (Hattori et al., 2012). Thus, we found a novel compound heterozygous mutation of *HAMP* in proband of 1‐III‐1 and a novel homozygous mutation in proband of 2‐II‐1. According to the Guideline of the American College of Medical Genetics and Genomics, the compound heterozygous and homozygous mutation led to type 1 diabetic‐onset JHH with autosomal recessive inheritance. In 1‐III‐1, due to normal blood coagulation function, the proband was not diagnosed as hemophilia A although he carried a pathogenic mutation in the *F8* gene.

In these Chinese *HAMP*‐JHH, insulin or other antidiabetic agents alone cannot control blood glucose due to progressive decline of C‐peptide; however, the concentration of C‐peptide increased after phlebotomy and deferoxamine treatment in 1‐III‐1 (Figure 3b). Diabetic onset of JHH with positive pancreatic autoimmune antibody was first reported worldwide. In these two cases, hyperglycemia can be resulted from iron deposition in pancreas. The excess iron facilitates production of free radicals to quench antioxidants (Kom et al., 2006). The ferrous iron catalyzes the formation of reactive hydroxyl radicals to get ferric iron, which is closely related with wide damage to pancreatic islet cell biological structures, including nucleic acids, cell membranes, and proteins (Merkofer et al., 2006; Wood, 2004). The process of pancreatic islet cell damage might bring about autoimmune disorder to illuminate why the 1‐III‐1 had ICA and IA–2A. The presence of antibody aggravates destruction of islet cell (Knip & Siljander, 2016). It proves iron deposition leading to pancreatic antibody existing and the treatment of iron overload could rescue islet cell function. Besides diabetes, treatments of iron chelation and phlebotomy rescued pancytopenia, serum ferritin (Figure 3c), and erectile function in 1‐III‐1.

*HAMP*‐JHH could cause a poor prognosis with cardiac or liver involvement. Differential diagnosis of JHH with type 1 diabetes at the early stage could improve the prognosis. 1‐III‐1 did not have obvious symptoms of cardiac involvement at the early stages. The sign of elevated liver enzyme was also neglected at the beginning in 2‐II‐1. In progressive stage, increased transferrin saturation can lead to the formation of abnormal forms of iron in plasma, such as non‐transferrin bind iron (NTBI) (Hershko et al., 1978). NTBI has specific kinetics contributing to cell toxicity, including cardiomyocytes and liver cells (Brissot et al., 2018). L‐type calcium channels play a role in NTBI uptake by cardiomyocytes which brings heart failure (Oudit et al., 2003). Although the survival time from onset of diabetes to death was quite different in the two cases, they all worsened with mere deferoxamine and died of major organ failure without venesection. Once diagnosed as JHH, the latent damage of target organs should be evaluated and monitored carefully and vital organs should be protected from iron deposition as early as possible. Phlebotomy can remove excess iron from the body (Brissot et al., 2018), as seen in the 1‐III‐1 where phlebotomy was more effective than subsequent iron chelation agents given over 2 months. The reported 16 cases (Table S2) were treated by periodic phlebotomies, and only one case was dying (Camaschella et al., 1997). Of note, periodic phlebotomies were more effective in treating iron overload than iron chelation agents.

In this study, we explored and reported the overall clinical management, including early stage of misdiagnosis, exact clinical and genetic diagnosis, treatment, and prognosis, of two novel atypical diabetic onsets JHH cases with novel compound heterozygous and homozygous mutation in the *HAMP* gene. It is first time that the positive pancreatic islet autoantibodies featured type 1 diabetic‐onset hemochromatosis. Through the review of two Chinese cases and worldwide published patients with pathogenic *HAMP* mutations, we attached the importance that the early differential diagnosis, evaluation, surveillance, and protection of crucial organs from iron deposition and proper phlebotomy at the early stage are crucial for the good prognosis in *HAMP*‐JHH patients.

## CONFLICT OF INTEREST

The authors declare that there is no conflict of interests regarding the publication of this paper.

## AUTHOR CONTRIBUTIONS

All authors participated sufficiently in the work to take public responsibility for the appropriateness of the collection, analysis, interpretation of the data, and editing the paper. HX Wu, JY Liu, DW Yan, and HD Zhou, designed the study protocol, contributed to the diagnosis and discussion of the study. HX Wu and HD Zhou wrote the manuscript. XH Zhuang, JY Liu, DW Yan, HX Wu, and HY Li provided and concluded clinical information. L Li performed the sequence analysis. DW Yan, HY Li, and ZG Zhou contributed to reviewing and editing the manuscript. HD Zhou is the guarantor and supervisor of this work. All authors read and approved the final version of the manuscript.

## Supporting information

Fig S1‐Table S1‐S2
