# Supplementary material for: Atypical juvenile hereditary hemochromatosis onset with positive pancreatic islet autoantibodies diabetes caused by novel mutations in HAMP and overall clinical management
Source: Mol Genet Genomic Med. 2020 Oct 5;8(12):e1522. doi: 10.1002/mgg3.1522 (PMC7767552; doi:10.1002/mgg3.1522)
Supplement: Supplementary file 1 — Fig S1‐Table S1‐S2 [file MGG3-8-e1522-s001.docx]

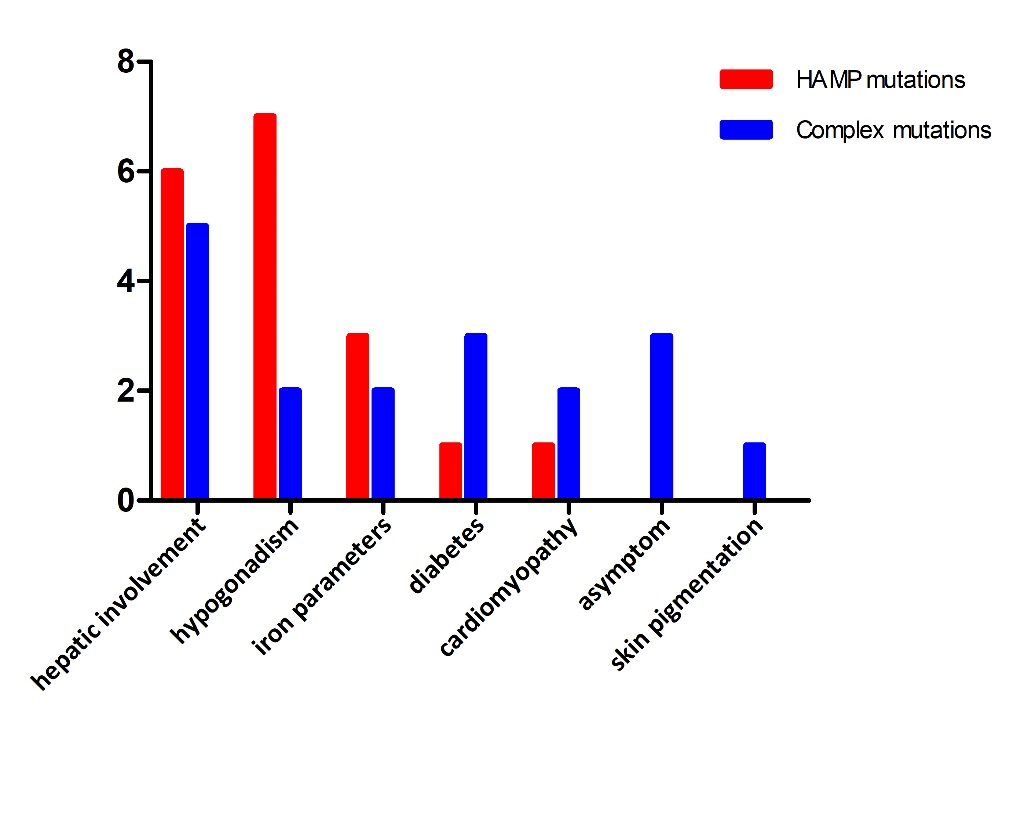


**Supplementary Figure 1** Analysis of *HAMP*-JHH symptoms at the onset between mere HAMP mutation and HAMP along with HEF or TRF2 mutations (complex mutations).

Fisher exact test was applied for HAMP and Complex mutations group, *p=0.211,* no statistical significance.

**Supplementary Table 1** Echocardiogram data from 1-Ⅲ-1

| Parameter Date | Sep. 2017 | Jan. 2018 |
| --- | --- | --- |
| LA, mm | 35 | 46 |
| LVD, mm | 49 | 56 |
| LVs, mm | 34 | 47 |
| RV, mm | 21 | 27 |
| PA, mm | 20 | 25 |
| EF, % | 58 | 34 |
| FS, % | 31 | 17 |

**Supplementary Table 2** Characteristics of worldwide *HAMP*-JHH patients

| **Nationality** | **Gender** | **Age at onset** | **Pathogenic mutations** | **Symptoms at onset** | **Treatments** | **References** |
| --- | --- | --- | --- | --- | --- | --- |
| Italy | NM | <30 | HAMP p.G32Dfs | NM | NM | ([Roetto et al., 2003](#_ENREF_20)) |
| Italy | NM | <30 | HAMP p.R56X | NM | NM | ([De Gobbi et al., 2002](#_ENREF_5); [Roetto et al., 2003](#_ENREF_20)) |
| Italy | M | 9 | HAMP p.R56X | Hepatic involvement | phlebotomy | ([Camaschella et al., 1997](#_ENREF_3)) |
| Pakistan | M | 21 | HAMP p.R42Sfs | Hypogonadism, hepatic involvement | phlebotomy | ([Lok et al., 2009](#_ENREF_13)) |
| Northern Greek | F | 26 | HAMP p.G32Dfs | Hypogonadism | phlebotomy | ([Papanikolaou et al., 2002](#_ENREF_18)) |
| Northern Greek | F | 22 | HAMP p.G32Dfs | Hypogonadism | phlebotomy | ([Papanikolaou et al., 2002](#_ENREF_18)) |
| Portuguese | M | 29 | *HAMP* c.-25G>A,  HEF p.H63D | Diabetes, cardiomyopathy | phlebotomy, erythropoietin | ([Matthes et al., 2004](#_ENREF_15)) |
| Portuguese | F | 24 | *HAMP* c.-25G>A,  HEF p.H63D | Asymptom | No | ([Matthes et al., 2004](#_ENREF_15)) |
| Portuguese | M | 28 | *HAMP* c.-25G>A | Hepatic involvement, cardiomyopathy | NM | ([Mendes et al., 2009](#_ENREF_16)) |
| Italy | M | 11 | HAMP p.C70R | Hepatic involvement | phlebotomy | ([De Gobbi et al., 2003](#_ENREF_4); [Roetto et al., 2004](#_ENREF_19)) |
| Japan | M | 26 | HAMP p.R75X,  TRF2 p.I238M^▲^ | Hepatic involvement, iron parameters change | phlebotomy | ([Hattori et al., 2012](#_ENREF_10)) |
| Australia | M | 38 | HAMP p.C78T | Diabetes, iron parameters change, hepatic involvement, hypogonadism | phlebotomy | ([Delatycki et al., 2004](#_ENREF_7)) |
| Australia | F | 6 | HAMP p.C78T | iron parameters change, hepatic involvement | Phlebotomy, pain killer | ([Delatycki et al., 2004](#_ENREF_7)) |
| France | M | 37 | *HAMP* nc.-153 C>T, HEF p.C282Y | Hepatic involvement | phlebotomy | ([Island et al., 2009](#_ENREF_11)) |
| Italy | M | 50 | *HAMP* c.-72C>T, HEF p.C282Y/ H63D^■^ | Diabetes, hepatic involvement | phlebotomy | ([Biasiotto et al., 2004](#_ENREF_2)) |
| Italy | M | 33 | *HAMP* c.-72C>T, HEF p.C282Y/ H63D^■^ | Iron parameters change | NM | ([Biasiotto et al., 2004](#_ENREF_2)) |
| UK | M | 27 | *HAMP* c.IVS2+1(-G) ^▲^, HEF p.C282Y^▲^ | Cardiomyopathy, hepatic involvement diabetes, skin pigmentation, hypogonadism | phlebotomy | ([Merryweather-Clarke et al., 2003](#_ENREF_17)) |
| Italy | M | 48 | HAMP p.R59X^▲^, HEF p.H63D^▲^ | Asymptom | NM | ([Badar et al., 2016](#_ENREF_1)) |
| Brazil | F | 25 | *HAMP* g.47G>A, HEF p.H63D^▲^ | Hypogonadism | phlebotomy | ([Lescano, Tavares, & Santos, 2017](#_ENREF_12)) |
| Portuguese | F | 24 | *HAMP* c.-25G>A | Hypogonadism | phlebotomy | ([Faria et al., 2016](#_ENREF_8)) |
| Portuguese | M | 24 | *HAMP* c.-25G>A | Hypogonadism | phlebotomy | ([Faria et al., 2016](#_ENREF_8)) |
| Italy | M | 28 | HAMP p.C70R, HEF p.C282Y^▲^ | Hepatic involvement | Phlebotomies, deferoxamine | ([Majore et al., 2004](#_ENREF_14)) |
| Brazil | F | 29 | *HAMP* g.47G>A | Hypogonadism | NM | ([Fonseca et al., 2016](#_ENREF_9)) |
| Brazil | F | NM | Not test | NM | NM | ([Fonseca et al., 2016](#_ENREF_9)) |
| Brazil | M | NM | Not test | NM | NM | ([Fonseca et al., 2016](#_ENREF_9)) |
| Brazil | M | NM | *HAMP* c.IVS3+42G >A^▲^, HEF p.H63D^▲^ | NM | NM | ([de Lima Santos et al., 2010](#_ENREF_6)) |

NM, not mentioned; F, female; M, male; ^▲^ heterozygous mutation; ^■^ compound heterozygous mutation.

**Supplementary References**

Badar, S., Busti, F., Ferrarini, A., Xumerle, L., Bozzini, P., Capelli, P., . . . Girelli, D. (2016). Identification of novel mutations in hemochromatosis genes by targeted next generation sequencing in Italian patients with unexplained iron overload. *Am J Hematol, 91*(4), 420-425. doi:10.1002/ajh.24304

Biasiotto, G., Roetto, A., Daraio, F., Polotti, A., Gerardi, G. M., Girelli, D., . . . Camaschella, C. (2004). Identification of new mutations of hepcidin and hemojuvelin in patients with HFE C282Y allele. *Blood Cells Mol Dis, 33*(3), 338-343. doi:10.1016/j.bcmd.2004.08.002

Camaschella, C., Roetto, A., Cicilano, M., Pasquero, P., Bosio, S., Gubetta, L., . . . Gasparini, P. (1997). Juvenile and adult hemochromatosis are distinct genetic disorders. *Eur J Hum Genet, 5*(6), 371-375. Retrieved from <http://www.ncbi.nlm.nih.gov/pubmed/9450181>

De Gobbi, M., Caruso, R., Daraio, F., Chianale, F., Pinto, R. M., Longo, F., . . . Camaschella, C. (2003). Diagnosis of juvenile hemochromatosis in an 11-year-old child combining genetic analysis and non-invasive liver iron quantitation. *Eur J Pediatr, 162*(2), 96-99. doi:10.1007/s00431-002-1114-6

De Gobbi, M., Roetto, A., Piperno, A., Mariani, R., Alberti, F., Papanikolaou, G., . . . Camaschella, C. (2002). Natural history of juvenile haemochromatosis. *Br J Haematol, 117*(4), 973-979. doi:10.1046/j.1365-2141.2002.03509.x

de Lima Santos, P. C., Pereira, A. C., Cancado, R. D., Schettert, I. T., Hirata, R. D., Hirata, M. H., . . . Guerra-Shinohara, E. M. (2010). Hemojuvelin and hepcidin genes sequencing in Brazilian patients with primary iron overload. *Genet Test Mol Biomarkers, 14*(6), 803-806. doi:10.1089/gtmb.2010.0056

Delatycki, M. B., Allen, K. J., Gow, P., MacFarlane, J., Radomski, C., Thompson, J., . . . Samuels, M. E. (2004). A homozygous HAMP mutation in a multiply consanguineous family with pseudo-dominant juvenile hemochromatosis. *Clin Genet, 65*(5), 378-383. doi:10.1111/j.0009-9163.2004.00254.x

Faria, R., Silva, B., Silva, C., Loureiro, P., Queiroz, A., Fraga, S., . . . Faustino, P. (2016). Next-generation sequencing of hereditary hemochromatosis-related genes: Novel likely pathogenic variants found in the Portuguese population. *Blood Cells Mol Dis, 61*, 10-15. doi:10.1016/j.bcmd.2016.07.004

Fonseca, P. F., Cancado, R. D., Uellendahl Lopes, M. M., Correia, E., Lescano, M. A., & Santos, P. C. (2016). HAMP Gene Mutation Associated with Juvenile Hemochromatosis in Brazilian Patients. *Acta Haematol, 135*(4), 228-231. doi:10.1159/000444119

Hattori, A., Tomosugi, N., Tatsumi, Y., Suzuki, A., Hayashi, K., Katano, Y., . . . Wakusawa, S. (2012). Identification of a novel mutation in the HAMP gene that causes non-detectable hepcidin molecules in a Japanese male patient with juvenile hemochromatosis. *Blood Cells Mol Dis, 48*(3), 179-182. doi:10.1016/j.bcmd.2012.01.002

Island, M. L., Jouanolle, A. M., Mosser, A., Deugnier, Y., David, V., Brissot, P., & Loreal, O. (2009). A new mutation in the hepcidin promoter impairs its BMP response and contributes to a severe phenotype in HFE related hemochromatosis. *Haematologica, 94*(5), 720-724. doi:10.3324/haematol.2008.001784

Lescano, M. A., Tavares, L. C., & Santos, P. (2017). Juvenile hemochromatosis: HAMP mutation and severe iron overload treated with phlebotomies and deferasirox. *World J Clin Cases, 5*(10), 381-383. doi:10.12998/wjcc.v5.i10.381

Lok, C. Y., Merryweather-Clarke, A. T., Viprakasit, V., Chinthammitr, Y., Srichairatanakool, S., Limwongse, C., . . . Robson, K. J. (2009). Iron overload in the Asian community. *Blood, 114*(1), 20-25. doi:10.1182/blood-2009-01-199109

Majore, S., Binni, F., Pennese, A., De Santis, A., Crisi, A., & Grammatico, P. (2004). HAMP gene mutation c.208T>C (p.C70R) identified in an Italian patient with severe hereditary hemochromatosis. *Hum Mutat, 23*(4), 400. doi:10.1002/humu.9232

Matthes, T., Aguilar-Martinez, P., Pizzi-Bosman, L., Darbellay, R., Rubbia-Brandt, L., Giostra, E., . . . Beris, P. (2004). Severe hemochromatosis in a Portuguese family associated with a new mutation in the 5'-UTR of the HAMP gene. *Blood, 104*(7), 2181-2183. doi:10.1182/blood-2004-01-0332

Mendes, A. I., Ferro, A., Martins, R., Picanco, I., Gomes, S., Cerqueira, R., . . . Faustino, P. (2009). Non-classical hereditary hemochromatosis in Portugal: novel mutations identified in iron metabolism-related genes. *Ann Hematol, 88*(3), 229-234. doi:10.1007/s00277-008-0572-y

Merryweather-Clarke, A. T., Cadet, E., Bomford, A., Capron, D., Viprakasit, V., Miller, A., . . . Robson, K. J. (2003). Digenic inheritance of mutations in HAMP and HFE results in different types of haemochromatosis. *Hum Mol Genet, 12*(17), 2241-2247. doi:10.1093/hmg/ddg225

Papanikolaou, G., Papaioannou, M., Politou, M., Vavatsi, N., Kioumi, A., Tsiatsiou, P., . . . Christakis, J. I. (2002). Genetic heterogeneity underlies juvenile hemochromatosis phenotype: analysis of three families of northern Greek origin. *Blood Cells Mol Dis, 29*(2), 168-173. doi:10.1006/bcmd.2002.0553

Roetto, A., Daraio, F., Porporato, P., Caruso, R., Cox, T. M., Cazzola, M., . . . Camaschella, C. (2004). Screening hepcidin for mutations in juvenile hemochromatosis: identification of a new mutation (C70R). *Blood, 103*(6), 2407-2409. doi:10.1182/blood-2003-10-3390

Roetto, A., Papanikolaou, G., Politou, M., Alberti, F., Girelli, D., Christakis, J., . . . Camaschella, C. (2003). Mutant antimicrobial peptide hepcidin is associated with severe juvenile hemochromatosis. *Nat Genet, 33*(1), 21-22. doi:10.1038/ng1053
